# Proactive health intervention outcomes in older adults with diabetes patients: a scoping review

**DOI:** 10.3389/fpubh.2026.1775618

**Published:** 2026-05-08

**Authors:** Xinyi Jin, Yao Cheng, Yating Ai, Xiaohong Zhang

**Affiliations:** 1 School of Nursing, Hubei University of Chinese Medicine, Wuhan, China; 2 Hubei Shizhen Laboratory, Wuhan, China; 3 Hubei Provincial Hospital of Traditional Chinese Medicine (The Affiliated Hospital of Hubei University of Chinese Medicine, Hubei Academy of Traditional Chinese Medicine), Wuhan, China; 4 Hubei Key Laboratory of theory and application research of liver and kidney in traditional Chinese medicine, Wuhan, China

**Keywords:** diabetes, older adults, health management, proactive health, scoping review

## Abstract

**Background:**

The phenomenon of global population ageing is driving a marked increase in the prevalence of diabetes mellitus among the older population, who face multiple health burdens requiring comprehensive management. Current models are limited by an inadequate focus on cultivating patients’ self-management capacities. Therefore, the establishment of a scientifically rigorous, holistic, proactive health management system covering the full disease cycle that encompasses the entire range of care for the designated population is essential. This study employs a scoping review methodology in order to systematically map and synthesize domestic and international evidence on proactive health interventions for older patients suffering from diabetes. The objective of this study is to elucidate the various intervention types, the core outcome measures, and the overall effect trends. The objective of this study is to comprehensively map the extant evidence and identify pivotal components, with the aim of informing the future development and implementation of proactive health management models for this group.

**Methods:**

This study adheres to the Arksey and O’Malley framework for scoping reviews. Systematic searches were conducted across both Chinese and English databases, including CNKI, WanFang, CQVIP, CBM, PubMed, the Cochrane Library, and Web of Science. The search covered publications from January 2015 to October 2025. Two independent researchers screened and extracted the data.

**Results:**

The search yielded 813 publications, of which 24 studies were included in the final analysis: 10 in English and 14 in Chinese. The included studies consisted primarily of 15 randomized controlled trials, 2 qualitative studies, 4 quasi-experimental studies, along with 2 cohort studies and 1 pilot study. Proactive health interventions are now analyzed through a dual framework of core components: educational empowerment, and behavioral support. The core outcome measures focused on physiological indicators, such as glycated hemoglobin and fasting blood glucose; patient-reported outcomes, such as quality of life and self-management capacity; and other health-related indicators, such as nutritional status and oral health. The literature includes research findings that indicate a positive trend across all measurement domains.

**Discussion:**

To optimize care for older adults with diabetes, implementation of adaptable proactive health management strategies is crucial. Evidence indicates that patient-centered models emphasizing active participation improve key outcomes like glycemic control, quality of life, and self-management. Their success depends on adaptability to resource levels and individual characteristics, supported by multidisciplinary teams delivering personalized, evidence-based plans. Further clinical translation requires expanded evidence from large-scale randomized controlled trials assessing long-term efficacy and applicability to older subgroups with comorbidities, alongside standardization of core elements under national or professional guidance to enhance system efficiency and patient outcomes.

## Introduction

The global population is experiencing age-related shifts in demographics, marked by an expanding older adults population (1). Amid this wave of aging, the rapid growth of the older adults diabetic population is particularly noteworthy (2). As demonstrated by data from the International Diabetes Federation (IDF): In 2019, the number of older adults with diabetes aged 65 to 99 worldwide reached 135.6 million, and is projected to increase to 276.2 million by 2045 (3). In China, this trend continues to manifest itself on an annual basis (4). Research has shown that China has over 78.13 million older adults with diabetes, the highest number globally (5). Older adults with diabetes frequently present not only with diabetes itself but also with a range of complications, along with varying degrees of cognitive dysfunction (6, 7). These factors lead to a significant decline in patients’ health-related quality of life (8, 9). Meanwhile, the ongoing medical costs required for disease management, along with productivity losses due to disability and premature death, impose a substantial economic burden on both patients’ families and society as a whole (10, 11). Therefore, establishing a scientifically sound, professionally accurate, and comprehensive health management system that encompasses the entire disease cycle for this population is crucial. However, the current health management model exhibits significant limitations. Primarily, there is a lack of systematic focus on developing patients’ self-management abilities, a critical factor often overlooked (12). Additionally, the evolution of supportive social infrastructure remains relatively underdeveloped, lacking comprehensive support systems ranging from medical guidance to community services (12, 13). These converging issues often leave patients passive in managing their chronic diseases, obstructing the development of sustainable self-management practices and negatively impacting overall treatment outcomes (14, 15).

In 2004, Hibbard et al. first systematically proposed the concept of “patient activation,” explicitly defining it as “an individual’s knowledge, skills, and confidence in managing their own health and healthcare affairs” (16). This pioneering research established the theoretical framework and measurement foundation for the concept, propelling patient activation into the formal international academic spotlight and laying important groundwork for subsequent advancements in patient-centered health management.

In 2020, researchers, including the Chinese scholar Li Xiangchen, established a clear definition of proactive health (17). They characterized it as a medical model that enhances human physiological functions or reverses chronic diseases by actively applying controllable stimuli to increase the body’s microscopic complexity and promote diversified adaptation. Its fundamental principles encompass preventative, precision-based, personalized, proactive, collaborative, shared, and self-regulated approaches (18, 19). Regarded as a key driver for advancing the “Healthy China” strategy, this innovative concept is a distinctive Chinese contribution to global health governance. However, as an emerging medical paradigm, its theoretical framework and practical pathways require ongoing refinement and development (20).

In 2022, an international journal elaborated on the concept of proactive health for the first time, framing it as a health service model centered on “proactive” characterized by prevention, precision, personalization, and collaborative sharing (21). With this international academic recognition and systematic articulation, proactive health has firmly secured its place on the global stage, bridging Chinese health philosophy with the discourse of international health management.

This scoping review was conducted in accordance with the methodological framework initially delineated by Arksey and O’Malley (22). This framework was subsequently refined by subsequent scholars (23, 24). The reporting of the review adhered to the PRISMA-ScR (Preferred Reporting Items for Systematic Reviews and Meta-analyzes extension for Scoping Reviews) guidelines (25). This scoping review aimed to systematically map evidence on active health management for older adults with diabetes by identifying relevant literature, clarifying key concepts and categories. Its iterative approach facilitates broad evidence mapping without quality assessment, providing a foundation for future systematic reviews to synthesize best practices.

## Methods

In accordance with the PRISMA-ScR five-step framework, the subject will be covered in full to ascertain all the relevant elements. This includes literature irrespective of the study design. The present study encompasses the following steps:

### Population

The study subjects are older patients aged 60 years and older who have been clinically diagnosed with diabetes. Eligible studies must explicitly identify this population as the primary participants.

### Concept

The core concept of this study is the intervention effect of “proactive health management” on older adults with diabetes and its evaluation methods. Proactive health management refers to a health service model centered on “proactive” characterized by prevention, precision, personalization, and collaborative sharing.

### Context

The implementation context of the study primarily involves community or hospital settings. Studies conducted exclusively in non-clinical environments (e.g., administration, finance, or purely academic/educational settings with no clinical component) or outside healthcare were excluded.

### Search strategy

Systematic retrieval was conducted across PubMed, Web of Science, Cochrane Library, the China National Knowledge Infrastructure (CNKI), VIP database, the Cumulative Index to Nursing and Allied Health (CINAHL), Wanfang database, and SinoMed. Searches employed a combination of subject headings and free-text terms, with a time frame spanning from January 2015 to October 2025. The search strategy was created based on the combination of Medical Subject Headings (MeSH) terms and keywords as follows: Aged, Aged 60 and over, Elderly, Aging, geriatric, Healthy aging, Active aging, Successful aging, Positive aging; Diabetes Mellitus, Type 1 Diabetes Mellitus, Type 2 Diabetes Mellitus, glycosuria, glycuresis; proactive health, active health, Positive Health, Automatic Health, Health Self-Care, Preventive Health, Self-Directed/Active Health Management, Health Promotion, Self-Health Care, Health management. Concurrently, relevant literature was manually retrieved to ensure comprehensiveness of included studies. Using PubMed as an example of English databases, the specific search strategy was as follows: #1 (“Aged” [Mesh] OR “Aged 60 and over” [Ti/Ab] OR “Older Adults” [Ti/Ab] OR “Seniors” [Ti/Ab]; OR “Elderly” [Ti/Ab] OR “Ageing” [MeSH] OR “geriatric” [Ti/Ab] OR “Healthy aging” [Ti/Ab], “Active aging” [Ti/Ab], OR “Successful aging” [Ti/Ab], OR “Positive aging” [Ti/Ab]); #2 (“Diabetes Mellitus” [MeSH] OR “Type 1 Diabetes Mellitus” [MeSH] OR “Type 2 Diabetes Mellitus” [MeSH] OR “glycosuria” [Ti/Ab], OR “glycuresis” [Ti/Ab]); #3 (“proactive health/active health” [Ti/Ab], “positive health” [Ti/Ab], “Automatic health” [Ti/Ab]OR “Preventive Health” [Ti/Ab] OR “Health Self Care” [Ti/Ab] OR “Self-Directed).

### Study screening and inclusion

All retrieved bibliographic records were imported into EndNote X21 reference management software. The screening process was conducted independently by two reviewers trained in evidence-based research methodology, divided into two stages:

Stage 1: Independently review titles and abstracts of all studies based on predefined inclusion and exclusion criteria, excluding those clearly ineligible. Studies with insufficient title/abstract information for determination were retained for Stage 2.

Stage 2: Full texts of studies retained in Stage 1 were obtained and underwent detailed assessment to determine final inclusion.

Any discrepancies between reviewers were resolved through discussion. If consensus could not be reached, a third reviewer was consulted for adjudication.

The inclusion criteria were studies that: ① Participants (P) participants were older adults who were aged 60 years or over and diagnosed with diabetes; ② Concept (C) involved the impact of providing proactive health management for older adults with diabetes; ③ Context (C) was primarily community or hospital settings; ④ The study designs included randomized controlled trials, quasi-experimental studies, cohort studies, qualitative studies and other original interventional or observational research.; ⑤ Literature types were original research; ⑥ Languages were Chinese or English.

The exclusion criteria were studies: ① Data repeated fewer than three times or without specification; ② Repeated analysis of the same indicator in the same cohort; ③ Non-publication in Chinese or English; ④ Conference proceedings and review articles; ⑤ Duplicate publications, incomplete data, or inaccessibility of full texts. In cases where the full text was not available through published sources, direct requests were made to the corresponding authors via email. Studies were excluded if the authors did not respond after two contact attempts.

### Data extraction

Data was extracted using a predefined data extraction sheet constructed for the purpose of this study. Data obtained from the full-text articles were organized according to the author(s) ID (author’s last name and year of publication), study design, country of origin of the study and so on. This data extraction and analysis was performed using Microsoft Excel.

As a scoping review aims to map the available evidence rather than to produce critically appraised and synthesized findings, and since the objective here was to obtain a broad overview of the contents and effects of active health interventions for older adults with diabetes, no formal quality appraisal of the included studies was performed.

## Results

### Literature screening results

**Figure 1:**
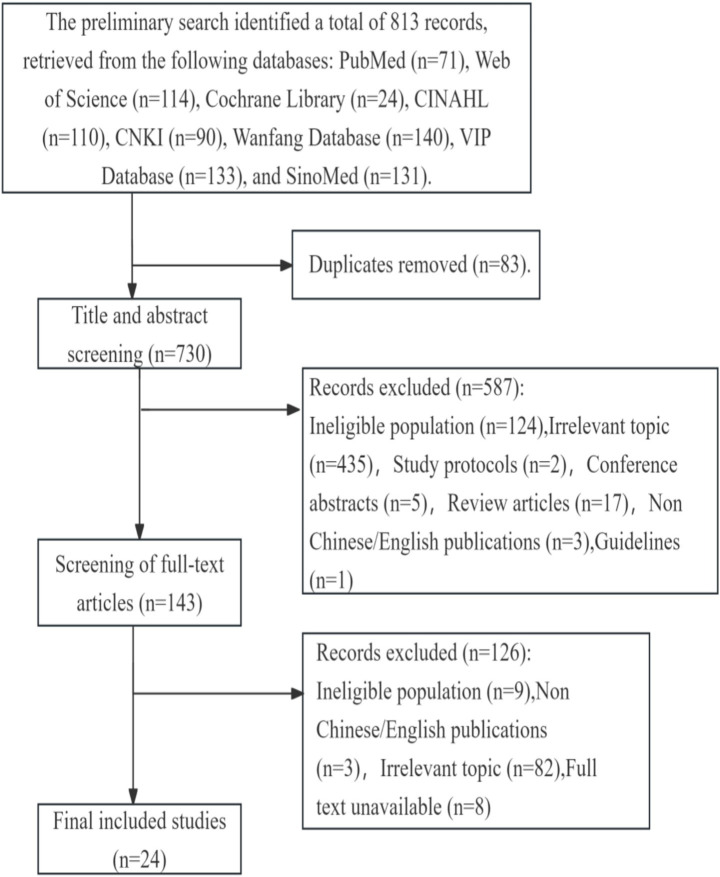
Flowchart of study retrieval and selection process (adapted from PRISMA).

After implementing the search strategy, the first stage of the selection process was carried out. An initial search yielded 813 publications. Following application of inclusion and exclusion criteria, 24 studies were ultimately retained. The screening process is illustrated in Figure 1.

### Basic characteristics of included literature

Of these, 10 were in English and 14 were in Chinese, with all publications occurring between 2015 and 2025. The study types comprised 15 randomized controlled trials (62.5%), and non-randomized controlled trials (37.5%), which includes quasi-experimental studies, cohort studies, and so on. The geographic scoping of the included studies was predominantly at the city level (*n* = 21), with fewer studies conducted at the state/province level (*n* = 3). The other key characteristics of the included studies are summarized in Table 1.

**Table 1:** General characteristics of the studies included in this research.

Included studies	Study type	Research setting	Sample size (*n*)		Duration and frequency of intervention	Measuring tools and details	Follow-up intensity and strategies
			Control group	Study group			
GaoHong xia (2019) (41)	RCT	Nantong (city)	36	36	11 months	Not mentioned	Not mentioned
Seangpraw Katekaew et al. (2023)(26).	Quasi-experimental Study	Phayao (city)	64	64	3 months, once a week for 180–240 min per session	Structured interview questionnaire	Two in-person follow-up visits and one telephone follow-up
HuWeidong et al. (2019) (27)	RCT	Jinan (city)	64	64	6 months	Mini nutritional assessment (MNA)	Not mentioned
JianQian Chao et al. 2015 (28)	RCT	Nanjing (city)	50	50	18 months At least once per month;	GRZ-120 body weight scale	Not mentioned
Yin Qiu Sheng (2015) (29)	Cohort study	Beijing (city)	Not applicable		5 years	Self-Administered Health Status and Cardiovascular Risk Factors Questionnaire	Annual health check-up with in-person follow-up
Hu Zhiqiang (2017) (30)	RCT	Foshan (city)	100	100	2 years	Not mentioned	Not mentioned
Huang Yanyun (2025) (42)	RCT	Nan’an (city)	83	97	1 year	GQOLI-74, ESGA, GHP questionnaire	WeChat platform follow-up once a week
Hisni, Dayan (2019) (43)	Pilot study	Cilegon (city)	6	6	6 weeks	PCCBQ, DDQHRI Questionnaire	Four in-person follow-up visits, plus two telephone follow-ups
					30–45 min per week		
Huang Yingqiu (2017) (31)	RCT	Chongqing (city)	92	92	1 year	Not mentioned	Not mentioned
Liu Fan et al. (2025) (32)	Quasi-experimental	Chengdu (city)	26	26	3 months	OHIP-14 scale, Turek modified method, 6S rRNA gene sequencing technology	Not mentioned
Mao Jin et al. (2018) (33)	RCT	Zunyi (city)	57	57	1 year	SF-36 health status questionnaire	Three times
Pu Peipei et al. (2020) (34)	RCT	Beijing (city)	356	356	10 months	Not mentioned	Not mentioned
Siti Khuzaimah Ahmad Sharoni (2017) (44)	Quasi-experimental Pilot Study	Selangor (state)	Not applicable		12 weeks	DFSBS, FCCS, Foot Care Outcome Expectations, Modified Brief Acceptability Scale	Weekly nurse visits during weeks 0–4, followed by biweekly visits during weeks 5–12.
Su Min (2019) (45)	RCT	Changde (city)	50	50	1 year	Not mentioned	Follow-up 6 months after discharge
Shi Jing et al. (2024) (46)	Quasi-experimental study	Nantong (city)	41	41	3 months	Diabetes Self-Management Behavior Scale Diabetes-Specific Quality of Life Scale	Monthly follow-up with patients via phone or WeChat, with in-person visits arranged by the management team as needed.
Yerdengqikeke (2018) (35)	RCT	Xinjiang (city)	100	100	20 months	Not mentioned	Not mentioned
Pormehr Sahar et al. (2023) (36)	RCT	Ahvaz (city)	20	20	1 month	WEMWBS, Subjective Vitality, Diabetes Quality of Life Brief Clinical Scale	Before baseline, after 8 training sessions
					90 min each time, twice a week		
Yu Haiyan et al. (2019) (37)	RCT	Hefei (city)	40	39	6 months	HAQ, SAS, SDS questionnaire	Not mentioned
Li Liuyi (2023) (38)	qualitative study	Chongqing (city)	Not applicable		11 interventions	SDSCA Scale and Semi-Structured Interview	Not mentioned
HaiBin Wu et al. (2022) (47)	RCT	Shenzhen (city)	50	50	12 months	Disease Control Effectiveness Assessment Tool and WHOQOL-BREF questionnaire	Not mentioned
Zheng Jieling et al. (2024) (48)	RCT	Foshan (city)	60	60	14 months	Self-developed awareness and compliance survey questionnaire, QOL scale	Not mentioned
Cathy M Murray (2016) (39)	Cohort Study	Ontario (province)	Not applicable		Not mentioned	Ontario Drug Benefit Program Database and Ontario Health Insurance Plan Physician Services Claims Database	Not mentioned
Wang XinYe et al. (2021) (49)	RCT	Changchun (city)	63	63	1 year	Continuous Glucose Monitoring System (CGMS), SAS, SDS, SF-36 Questionnaire	Dynamic implementation of home visits
Mohammad Y Alkawaldeh (2020) (40)	Qualitative study	Massachusetts (state)	Not applicable		30 days	Semi-structured interview guide and General Characteristics Questionnaire	Semi-structured in-depth interviews, average duration of 45 min, totaling 11 sessions

Included studies	Intervention		Intervention outcome measures	Post-intervention effects
	Control group	Study group		
Gao Hongxia (41)	Routine Care	Health Management Based on the Zhixinxing Model	AD	The glycemic control rate in the observation group was 97.22%
Seangpraw Katekaew et al. (26)	Regular health advice and health diary from the area’s local sub-district health promotion hospital	Based on the guidelines, the health literacy-based curriculum employed researcher-developed manuals and diaries to guide participants in the daily recording and practice of self-management behaviors.	ACDHKL	Fasting blood glucose (FBG) decreased by 9.8 units, HbA1c (decreased by 0.4 units), estimated glomerular filtration rate increased by 14.0 units, diabetes health literacy improved by 3.1 points, self-efficacy increased by 2.9 points, and self-care behavior improved by 10.4 points.
Hu Weidong et al. (27)	Routine Chronic Disease Management	Community Health Management	E	Nutritional status score: 28.12 ± 3.42 vs. 24.16 ± 2.68 (16.4% increase). showed a significantly higher value (*p* < 0.05)
JianQian Chao et al. (28)	Routine Care	Comprehensive Health Management Model	H	BMI (*p* = 0.004), and FBG (*p* = 0.042).
Yin Qiu Sheng (29)	The program provides a comprehensive management service through the following means: lectures, personalized follow-up, stepwise medication, and lifestyle guidance.		DST	The rate of achieving target FBG levels increased by 16.38%, and the rate of achieving target total cholesterol levels increased by 15.42%. Both differences were statistically significant (*p* < 0.05).
Hu Zhiqiang (30)	Standard treatment and care	Community health management	CFIJ	Diabetes awareness rate 98.00% vs. 72.00% (36.1% increase); Control rate: 84.00% vs. 51.00% (64.7% increase) Dietary adherence rate: 92.00% vs. 76.00% (21.1% increase). All differences were statistically significant (*p* < 0.05).
Huang Yanyun (42)	Routine care	Health promotion behavioral care	BK	Quality of life improved from 4.2 to 37.3% (*p* < 0.001); ESCA score: 115.36 ± 4.70 vs. 103.23 ± 2.19 (11.7% increase); GHP score: 75.20 ± 4.31 vs. 68.66 ± 3.03 (9.5% increase). Both differences were statistically significant (*p* < 0.05).
Hisni, Dayan (43)	Usual care	The 5A’s self-management support program	HLP	PCCBQ score: 92.83 ± 13.17 vs. 58.17 ± 6.08 (increase of 59.6%); FBG 132.00 ± 28.86 vs. 223.00 ± 77.43 (40.8% decrease); Total cholesterol: 145.67 ± 32.84 vs. 190.00 ± 41.62 (23.3% decrease); HDL cholesterol: 52.17 ± 7.19 vs. 41.50 ± 3.39 (*p* = 0.00, 25.7% increase); LDL cholesterol: 56.83 ± 7.88 vs. 93.00 ± 35.91 (*p* = 0.07, 38.9% decrease); Systolic blood pressure: 131.67 ± 13.29 vs. 148.00 ± 18.34 (11.0% decrease); Diastolic blood pressure: 72.67 ± 3.32 vs. 88.33 ± 4.08 (decrease of 17.7%). All differences were statistically significant (*p* < 0.05).
Huang Yingqiu (31)	Routine community care	Community health management	CL	BMI 22.9 ± 2.3 vs. 24.8 ± 2.0 (7.7% decrease); FBG 6.8 ± 0.9 vs. 7.7 ± 1.2 (11.7% decrease); 2hPBG 8.2 ± 0.7 vs. 11.6 ± 1.1 (29.3% decrease); HbA1c 6.3 ± 0.8 vs. 7.2 ± 1.0 (12.5% decrease). All differences were statistically significant (*p* = 0.000).
Liu Fan et al. (32)	Routine oral care	Oral health promotion interventions	AC (Oral)	Plaque Index (PLI) significantly decreased (1.73 → 1.33), oral health-related quality of life (OHIP-14 score 6.21 → 0.41) and mastery of oral health knowledge (0.63 → 0.94) significantly improved;
Mao Jin et al. (33)	Routine clinical management	Implementation of health promotion clinical management model	B	The SF-36 total score increased from 65 to 78 points (20% increase, p < 0.05),
Pu Peipei et al. (34)	Routine management	Community health management	CGM	2hPBG decreased from 11.2 mmol/L to 9.2 mmol/L (a reduction of 17.9%, *p* < 0.05) and Glycemic control rate increased from 48 to 73% (an increase of 52.1%, *p* < 0.05) were statistically significant (*p* < 0.05).
Siti Khuzaimah Ahmad Sharoni (44)	Comprehensive educational program based on self-efficacy theory		ABCKL (foot)	Self-care behaviors: 69.00 vs. 45.00 (53.3% improvement); Foot care self-efficacy: 44.00 vs. 30.00 (46.7% improvement); Nursing knowledge score: 11.00 vs. 8.00 (37.5% increase); FBG 6.10 mmol/L vs. 7.90 mmol/L (22.8% decrease); Quality of life physical symptom score: 14.00 vs. 23.00 (39.1% decrease). All differences were statistically significant (*p* < 0.05).
Su Min (45)	Standard care	Health management intervention	CDKP	FBG 6.07 ± 2.32 vs. 9.02 ± 2.17 mmol/L (32.7%decrease), 2hPBG 9.01 ± 1.17 vs. 11.28 ± 2.65 mmol/L (20.1% decrease), and HbA1c 6.97 ± 2.24% vs. 9.85 ± 2.03% (29.2% decrease). disease control 1.89 ± 0.35 vs. 1.59 ± 0.27 points (18.9% increase) and self-management 4.20 ± 0.39 vs. 3.61 ± 0.69 points (16.3% increase). All differences were statistically significant (*p* < 0.05).
Shi Jing et al. (46)	Routine care	The ‘Internet Plus’ healthcare management model	BCK	FBG 7.12 ± 1.03 vs. 10.22 ± 1.57 (30.3% decrease); 2hPBG 9.08 ± 1.12 vs. 14.13 ± 1.64 (35.7% decrease) showed statistically significant differences (*p* < 0.05 for all). Self-management capabilities increase 53.6–74.6% and quality of life decrease 50.3–41.5% showed statistically significant differences (*p* < 0.001 for all).
Yerdengqikeke (35)	Routine community management	Integrated community health management	C	BMI 22.94 ± 2.31 vs. 24.86 ± 2.33 (*p* < 0.001, 7.7% decrease); FBG 6.81 ± 0.93 vs. 7.79 ± 1.34 (*p* < 0.001, 12.6% decrease); 2hPBG 8.24 ± 0.79 vs. 11.68 ± 1.16 (*p* < 0.001, 29.5% decrease).
Pormehr Sahar et al. (36)	Join the training waiting list	Participate in health promotion lifestyle training	BGO	Mental health score: 39.90 ± 4.80 vs. 33.85 ± 4.81 (*p* < 0.001, 17.9% increase); Quality of life score: 35.00 ± 6.13 vs. 29.15 ± 4.67 (*p* < 0.002, 20.1% increase); Subjective vitality score: 23.00 ± 3.31 vs. 17.80 ± 3.15 (*p* < 0.001, 29.2% increase).
Yu Haiyan et al. (37)	Conventional drug therapy	APP-based health management plan	BC	FBG 7.75 ± 1.95 vs. 8.31 ± 1.95 (6.7% decrease); 2hPBG 9.01 ± 2.27 vs. 9.75 ± 3.67 (7.6% decrease); Triglycerides: 1.1 ± 0.81 vs. 2.1 ± 0.98 (47.6% decrease); SAS Score: 45.23 ± 9.32 vs. 50.25 ± 7.95 (10.0% decrease); SDS score: 46.08 ± 8.58 vs. 55.80 ± 8.92 (17.4% decrease). All differences were statistically significant (*p* < 0.05).
Li Liuyi (38)	Study objectives: redefining awareness, strengthening behavior management, and regulating negative emotions in diabetes.		CGK	FBG have dropped to normal ranges, with self-management scores improving by 21 points on average.
HaiBin Wu et al. (47)	Routine treatment and management	Health management through traditional Chinese medicine principles	BP	Disease control efficacy of 94.00% vs. 74.00% (*p* = 0.006, 27.0% improvement); Quality of life improved by 8.8 to 16.2% (*p* < 0.001)
Zheng Jieling et al. (48)	Standard care	PDCA (Plan-Do-Check-Act) -based health management intervention model	ACF	FBG 5.12 ± 0.34 vs. 6.23 ± 0.49 (17.8% decrease); 2hPBG 6.85 ± 1.07 vs. 8.59 ± 1.33 (20.3% decrease); HbA1c 6.63 ± 0.87 vs. 7.26 ± 1.14 (8.7% decrease); diabetes-related knowledge improved by 12.5 to 14.4% (*p* < 0.001)
Cathy M. Murray (39)	Routine diabetes care	Diabetes self-management education	DTUV	Statin use: 70.6% vs. 69.4% (1.7% increase); Anti-diabetic medication use: 83.7% vs. 82.0% (2.1% increase); Anti-hypertensive medication use: 90.2% vs. 89.7% (0.6% increase); Ophthalmology/optometry visit rate: 78.7% vs. 72.7% (3% increase). All differences were statistically significant (*p* < 0.05).
Wang XinYe et al. (49)	Routine care intervention	Family and organizational health education model	BCFGQ	FBG 6.7 ± 1.7 vs. 10.5 ± 1.9 (*p* < 0.001, 36.2% decrease); 2hPBG 8.2 ± 2.8 vs. 13.0 ± 2.6 (*p* < 0.001, 36.9% decrease); HbA1c 6.4 ± 0.5 vs. 7.2 ± 0.6 (*p* < 0.001, 11.1% decrease); Quality of life improved by 10.1.% to 15.8 (*p* < 0.001); Medication adherence rate: 55 vs. 61 (9.8% decrease); Diabetes knowledge mastery rate: 46 vs. 58 (20.7% decrease); Nursing satisfaction: 56 vs. 63 (*p* = 0.006, 11.1% decrease) All differences were statistically significant (*p* < 0.05).
Mohammad Y Alkawaldeh (40)	Using the tablet-based application ASSIST		AFJKL	Enhanced self-management discipline, improved awareness of blood glucose fluctuations, dietary choices, and medication necessity, along with increased positive attitudes and behaviors.

Appendix: A. Level of health knowledge acquisition, B. Quality of Life, C. Glycaemic Control Effectiveness, D. Glycaemic Target Achievement Rate, E. Nutritional Status, F. Adherence, G. Psychological State, H. Health Indicators, I. Diabetes Awareness Rate, J. Disease Treatment Rate, K. Patient Self-Care Capacity, L. Health behavior, M. Exercise Control, N. Dietary Control, O. Patient Vitality, P. Disease Control Effectiveness, Q. Hypoglycaemia Incidence Rate, R. Nursing Satisfaction, S. Lifestyle, T. Incidence of cardiovascular events, U. Drug utilization rate, V. Visit rate.

### Intervention components included in the study

#### Categories of intervention measures

**Figure 2:**
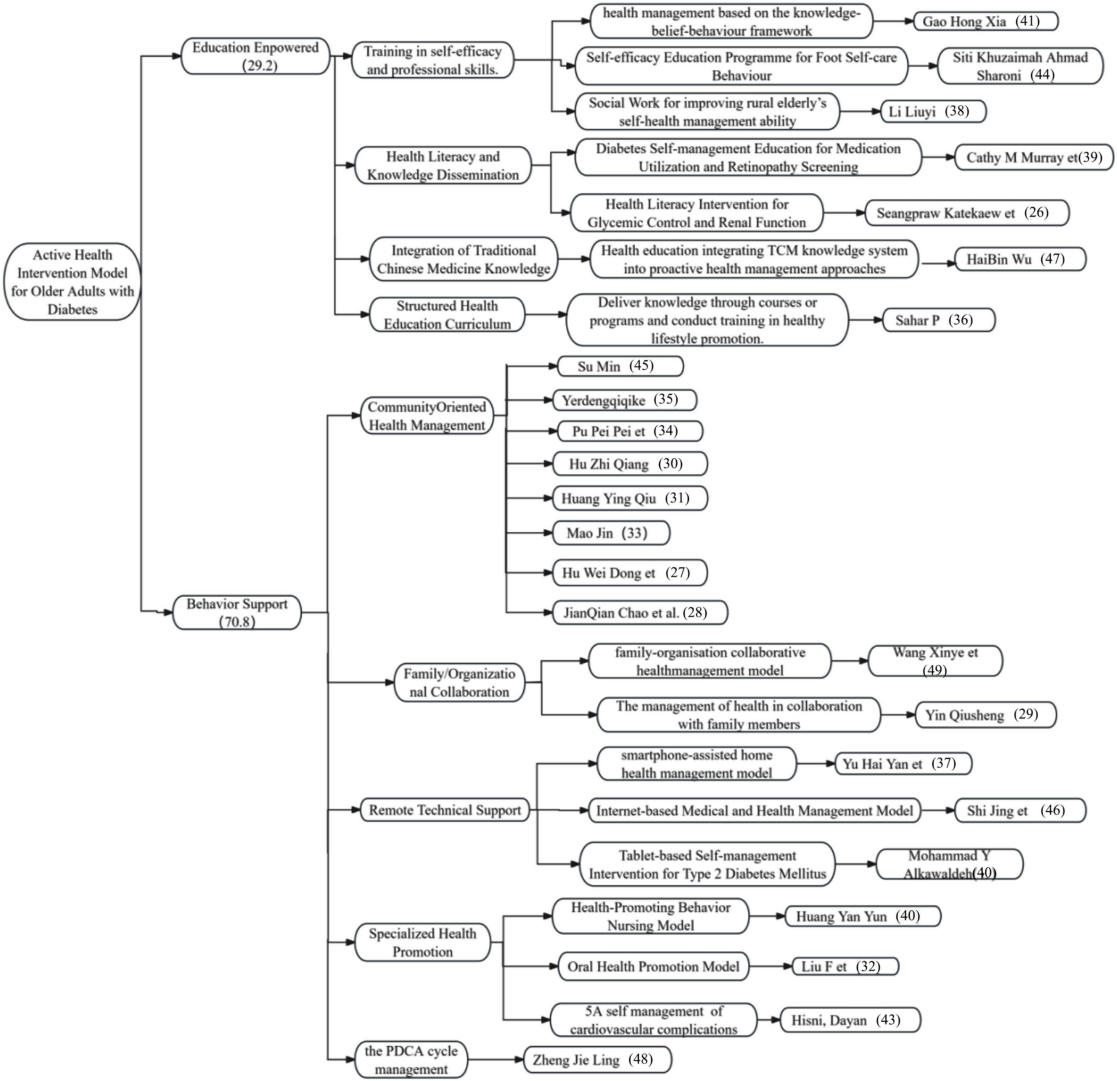
Distribution of Intervention Measures.

The extant literature on the subject indicates that proactive health intervention models for older chronic disease patients can be categorized into two types: educational empowerment and behavioral support. In this context, behavior support interventions are predominant, accounting for 70.8% of measures, while educational empowerment constitutes 29.2%. This distribution pattern is indicative of contemporary academia’s predilection for behavior-oriented intervention pathways, which may be attributable to the relative maturity of theoretical frameworks and practical applications within this field. The paucity of research in the field of educational empowerment suggests a significant potential for further research, innovation, and expanded application in this domain. For a more detailed overview, please refer to Figure 2.

#### Intervention setting and duration

With regard to the settings of intervention, the majority of the included studies were community-based (26–40), a figure that is more prevalent than hospital-based settings (41–49). With regard to the duration of intervention, the study protocols exhibited significant variation, with the following distribution: the majority of studies had a duration of 1 year or longer (28–31, 33, 35, 42, 45, 47–49); four studies had a duration of 6–11 months (27, 34, 37, 41); and seven studies had a duration of 3 months or less (26, 32, 36, 40, 43, 44, 46).

#### Evaluation indicators for active health management outcomes in older adults with diabetes

This scoping review indicates that contemporary proactive health management intervention systems for older adults with diabetes exhibit a diverse landscape, with “behavioral support” as the mainstream approach, complemented significantly by “educational-empowered” models, yielding positive outcomes across multiple dimensions.

Specifically, behavioral support, the prevailing intervention model (accounting for 70.8%) in contemporary proactive health management for older adults with diabetes, encompasses strategies including community management, PDCA cycle management, family collaboration, and remote technical support, while studies by Hu Weidong et al., Chao Jianqian, and Hu Zhiqiang confirmed the reliability and practicality of these strategies (27, 28, 30). Additionally, Hisni et al. implemented a 5A model-based cardiovascular self-management support program, which improved older adults with diabetes’ behavioral habits and clinical outcomes via structured intervention (43). Although limited research on remote technical support, representative studies like those by Yu Haiyan et al. and Shi Jing et al., which showed that such approaches could improve physiological indicators and quality of life (37, 46). Specifically, Shi Jing et al.’s Internet-based healthcare management model for older adults with diabetes enhanced glycemic control and patient satisfaction through real-time monitoring and personalized feedback, highlighting the potential of technology-driven strategies in boosting management efficiency and patient experience. Furthermore, the emergence of distinctive indicators such as family support level and Traditional Chinese Medicine constitution scores reflects the innovative integration of behavioral support and educational empowerment interventions in practice, deepening relevant research perspectives and specialized applications.

Interventions that are education-empowered play a pivotal role at specific cognitive levels. This assertion is evidenced by most literature reporting improvements in “health knowledge levels”, e.g., as demonstrated in studies such as Ahmad et al., whose self-efficacy education program for older diabetic patients in Malaysia significantly enhanced foot care knowledge through structured cognitive training (44). This collective evidence underscores the remarkable efficacy of education-based interventions in enhancing patient cognitive capabilities, particularly in translating knowledge into actionable self-care behaviors. Meanwhile, Li Liuyi’s cognitive-behavioral theory-based social work group intervention (originated from the education-empowered category) for rural older patients in China enhanced glycemic control, self-management, and psychological well-being, validating the efficacy of integrated community approaches in underserved rural areas (38).

The present intervention system is multi-faceted in nature, incorporating behavioral and educational components. It has been demonstrated to have comprehensive and robust evidence of effectiveness in improving key health outcomes among older adults with diabetes. These outcomes include physiological, behavioral, psychological, and quality of life aspects. The effectiveness is said to be achieved through synergistic strategies. For a more detailed analysis, please refer to Table 2.

**Table 2:** Evaluation indicators for active health management outcomes in older adults with diabetes.

Outcome domain	Specific Indicator	Remarks
Physiological and biochemical indicators	Glycated hemoglobin (HbA1c)	Improved: (26, 31, 45, 48, 49) The decrease ranged from 8.7 to 29.2% No significant improvement (*p* > 0.05) (32, 37) Only mentions blood sugar control without specifying that it refers to glycated hemoglobin (41). Significant differences in diabetes control dimensions indicating that FPG, 2hPG, and HbA1c levels decreased to within the normal range; or that the reduction in FPG, 2hPG, and HbA1c levels exceeded 20% compared to pre-treatment levels, though this was not explicitly stated (30).
	Fasting blood glucose (FBG)	Significant improvement: (26, 29, 31, 35, 37, 38, 43, 45, 46, 48, 49) The decrease ranged from 6.7 to 40.8%. As above (30, 41).
	2-h postprandial glucose (2hPG)	Significant improvement: Corresponding to a relative reduction of about 7.6 to 36.9% (31, 34, 35, 37, 45, 46, 48, 49). As above (30, 41).
	Blood pressure	Reported significant improvement, systolic blood pressure 11.0% decrease, diastolic blood pressure 17.7% decrease (43). Diastolic blood pressure showed significant improvement (*p* = 0.019), while systolic blood pressure showed no significant improvement (*p* = 0.821) (28). No significant improvement (*p* = 0.131) (29).
	Body mass index (BMI)	Reported improvement (28, 31, 35), no significant improvement (37) no significant improvement (*p* = 0.377) (47)
	Blood lipids	Total cholesterol decreased 47.6% (37). Significant differences were observed in total cholesterol (*p* = 0.028), but no significant differences were found in triglycerides (*p* = 0.370), LDL-C (*p* = 0.121), or HDL-C (*p* = 0.234) (29). Total cholesterol and HDL were significantly reduced (*p* < 0.05), whilst LDL showed no significant difference (*p* = 0.07) (43).
	Nutritional indicators	Reported significant improvement (27). Nutritional status scores increased from 18.65 ± 2.75 points to 28.12 ± 3.42 points (*p* < 0.05), representing a 16.4% improvement compared to the control group.
	Oral health	The plaque index (PLI) decreased from 1.73 ± 0.13 to 1.33 ± 0.14 (a reduction of 23.1%, *P* < 0.05), and the Oral Health Impact Profile (OHIP-14) score decreased from 6.21 to 0.41 (a reduction of 93.4%, *p* < 0.001) (32).
Quality of life and psychological indicators	Quality of Life	Reported significant improvement (33, 36, 37, 42, 44, 46, 48, 49). Quality of life scores improved by 20.1 to 39.1% overall, SF-36 score improved 20%. Some studies reported dimensions increases ranging from 4.2 to 50.3%. Significant reduction in physiological symptom dimension scores (23.00 → 14.00) (46). No significant decline in psychosocial functioning dimension (median baseline 15.00 → post-intervention 26.00) (*p* > 0.05).
	Depression/Anxiety Scores	Reported significant improvement (49) SAS anxiety scores decreased from 56.99 ± 5.26 to 23.15 ± 4.27 (a reduction of 59.4%, P < 0.001) HAQ scores (*P* > 0.05), but SAS and SDS (*p* < 0.05) (37).
	Psychological wellbeing	Reported significant improvement (17.9% increase) (36). As this is a qualitative study, while it indicates significant impact, there is no data to support this claim (38, 40).
Behavioral indicators	Self-management ability/behavior	Reported significant improvement (9.5–59.6%) (26, 42–46). Each dimension improved from 53.6 to 74.6% (46). As above (38, 40).
	Patient adherence	Reported significant improvement (improve 0.6%–9.8) (39, 49) As above (38). Medication adherence: Significant increases were observed for statins, antidiabetic drugs, and anti-hypertensive medications, while increases for proton pump inhibitors (27.1% vs. 26.8%, *p* = 0.2) and levothyroxine (16.6% vs. 16.1%, *p* = 0.07) were not significant (48).
	Health literacy	Reported significant improvement (26, 31, 41, 48)
	Dietary/exercise behavior	Reported significant improvement (26, 28, 30, 31, 34, 46). As this is a qualitative study, while it indicates significant impact, there is no data to support this claim (40).

## Discussion

Theoretical frameworks and empirical findings suggest that this management approach, which organically integrates two intervention modes—educational empowerment and behavioral support—could potentially lead to synergistic effects. This possibility warrants further investigation in robust trials. The educational empowerment intervention, grounded in the “knowledge–Belief–Behavior” theoretical framework, lays the cognitive foundation for behavioral change by enhancing health promotion and self-efficacy (26, 36). The behavioral support intervention employs structured strategies such as the PDCA cycle and family collaboration to provide external support for behavior maintenance (48, 49). These two interventions form a temporal sequence of “cognitive enlightenment - behavior establishment - Habit Maintenance” intervention continuum. Spatially, they establish a multi-tiered support network spanning “Individual–Family–Community.” As proposed in the WHO Global Report on Diabetes 2023, multi-modal integrated interventions significantly outperform single-intervention approaches (50).

Concurrently, research findings indicate a bidirectional relationship between improvements in the physical well-being of older patients with diabetes and enhancements in their quality of life. Enhanced physiological indicators like blood glucose control directly alleviate disease symptoms and strengthen physical function, laying a physiological foundation for improved quality of life (48). Conversely, enhanced quality of life—particularly psychological well-being—significantly improves treatment adherence and self-management initiative, thereby optimally regulating physiological indicators (36). This positive physiological-psychological cycle was thoroughly validated in Seangpraw et al.’s health literacy intervention and Ahmad et al.’s self-efficacy program (26, 44). Furthermore, other studies also confirmed the bidirectional regulatory effect between psychological state and glucose metabolism indicators in a longitudinal study of older diabetic populations (51, 52). Also, some research further indicated that positive psychological experiences can increase medication adherence by over 30% in older adults with diabetes, serving as a crucial safeguard for sustained improvement in physiological indicators (53).

Concurrently, the synergistic adaptation mechanism between individualization and standardization is a crucial prerequisite for achieving synergistic effects in proactive health management. The organic integration of personalized health guidance based on Traditional Chinese Medicine constitution theory with standardized community health management processes ensures the scientific rigor and standardization of interventions through a standardized framework while enhancing the adaptability of intervention protocols to specific populations through individualized adjustments.

Despite the preliminary indications from extant research that proactive health management exerts a synergistic role in the prevention and treatment of diabetes among the older, the studies identified in this review are still characterized by several significant limitations. While behavioral support is the dominant intervention, research on technological support demonstrates a notable gap, which is in stark contrast to the broad prospects of digital health in the management of chronic diseases. The fundamental cause of this disparity can be attributed to the inadequate accessibility of technological support for older patients and the absence of age-appropriate design (54). The digital divide remains pronounced, with many older adults facing challenges of low device accessibility and limited digital literacy. Concurrently, existing health applications or wearable devices frequently fail to adequately account for the physiological and cognitive characteristics of older adults. These include overly small fonts, complex operations, and poor adaptability to declining vision and hearing. The result is often a sub-optimal user experience and compliance (55, 56). Consequently, future research must progress beyond superficial discussions of innovation potential to undertake in-depth explorations of the development of age-friendly technological solutions that are tailored to the physiological and psychological characteristics of older adults. In addition, a cost-effectiveness analysis and an evaluation of the practical outcomes of integrating these solutions with educational and behavioral support measures should be conducted.

Second, while most studies involved intervention periods exceeding 1 year, long-term follow-up data on outcomes after intervention cessation is generally lacking. It remains unclear whether patients’ self-management motivation, improved physiological indicators, and quality of life can be sustained once external professional support diminishes or ceases (57, 58). Research indicates that only 14% of digital health intervention reviews included long-term follow-up results. Furthermore, when external support ceases, the retention rate of self-management behaviors among older patients drops to 30–50%, with improvements in physiological indicators declining by 40–60% (59). This suggests that future proactive health management models must prioritize “sustainability” as a core consideration. Improvement strategies should include: establishing mechanisms for a smooth transition from professional guidance to peer support and family empowerment; exploring feasible pathways to leverage existing community resources for sustaining intervention outcomes; and conducting research incorporating long-term follow-up and health economic evaluations. This will assess their true public health value and resource allocation requirements for large-scale implementation.

Furthermore, despite the widespread promotion of “multidisciplinary teams” and “standardization,” their specific connotations remain relatively ambiguous. Future research and practice should propose differentiated implementation pathways based on distinct intervention scenarios and dominant models to enhance their operability and effectiveness.

Finally, methodological limitations in the studies may affect the comprehensiveness of conclusions. As “proactive health management” lacks a unified MeSH subject heading, this review primarily relied on keyword searches, potentially resulting in literature omissions (60). Furthermore, the included studies were predominantly conducted by Chinese researchers. While providing valuable local insights, this limits the generalizability of conclusions across different cultural contexts and healthcare systems (61). Future efforts should strengthen international collaboration, conduct cross-cultural comparative studies, and promote the international standardization of concepts and measurement tools related to proactive health management.

### Innovation and limitations

Despite the absence of a prospective registration, the academic innovation of this study is unmistakable and conspicuous. The existing literature is devoid of research that systematically reviews and integrates active health interventions for older adults with diabetes using a comprehensive scoping review methodology. In consideration of the aforementioned context, the fundamental contributions of this study are evident at three distinct levels: Firstly, the text provides empirical evidence demonstrating the superiority of active health management over traditional care models in significantly improving the quality of life and key physiological indicators of older adults with diabetes. Secondly, it provides a systematic elucidation of the underlying mechanisms of this management model. The findings provide an evidence-based perspective for addressing the prevalent passive health management challenges among this population, thus laying a robust evidence foundation for advancing patient-centered proactive health models in this field.

Notwithstanding these contributions, it is essential to acknowledge the methodological limitations of this scoping review to ensure a balanced interpretation of the findings. First, the included studies predominantly reported favorable improvements in outcome measures, indicating potential positive result bias and publication bias. This may stem from the difficulty of publishing studies with negative or neutral results, as well as selective reporting of outcomes in some investigations. Such factors may compromise the objectivity and comprehensiveness of the evidence, potentially affecting the generalizability of this review’s conclusions. Second, the presentation of results primarily relies on count statistics and descriptive analysis. Consequently, it cannot provide a quantitative synthesis of intervention effects (such as effect sizes or risk ratios) or grade the quality of evidence, as systematic reviews do. This means that although we observe most studies reporting positive outcomes, we cannot determine the magnitude of their clinical significance or identify optimal implementation strategies. Finally, the majority of the “research settings” included in this study focused on specific healthcare institutions or communities at the urban level. This distribution indicates that the findings are difficult to generalize to rural or resource-poor areas, potentially overlooking the differentiated needs of older adults with diabetes across different regions. Consequently, there exists a significant geographical and contextual gap in the evidence base, which may exacerbate the risk of health service inequalities.

### Summary

In the context of district-level clinical treatment, the extant literature suggests that an active health management model centered on older adults with diabetes and emphasizing their active participation may offer potential benefits. The findings of this study indicate that the model is effective in improving key physiological indicators, such as blood glucose levels, enhancing patients’ quality of life, and strengthening their self-management capabilities. It is recommended that patients be integrated into systematic health management plans during clinical treatment. The key to success lies in the model’s ability to demonstrate high adaptability by leveraging clear resource tiers (city, state/provincial, or national) and incorporating individual patient characteristics to develop personalized intervention strategies. Practice has demonstrated that this approach effectively controls blood glucose while simultaneously enhancing patients’ quality of life and self-care capabilities. During interventions, multidisciplinary international expert teams can be formed to provide scientifically grounded, globally applicable, and individualized treatment plans.

To advance the proactive health management model for older adults with diabetes, future research should: design large-scale, multicenter randomized controlled trials at the national or cross-regional level to strengthen the evidence base; systematically conduct long-term (e.g., ≥5 years) clinical follow-ups to evaluate the model’s sustained impact on endpoint events, quality of life, and healthcare economics; validate the applicability of personalized models for older subgroups with complications such as cardiovascular disease and chronic kidney disease within specialized medical centers or community collaborative networks; and ultimately, under the leadership of national or professional societies, standardize core intervention elements and implementation processes to facilitate the standardized application and promotion of this model across different tiers of healthcare systems, including urban and community settings.
